# Functional importance of the oligomer formation of the cyanobacterial H^+^ pump *Gloeobacter* rhodopsin

**DOI:** 10.1038/s41598-019-47178-5

**Published:** 2019-07-24

**Authors:** Azusa Iizuka, Kousuke Kajimoto, Tomotsumi Fujisawa, Takashi Tsukamoto, Tomoyasu Aizawa, Naoki Kamo, Kwang-Hwan Jung, Masashi Unno, Makoto Demura, Takashi Kikukawa

**Affiliations:** 1 Faculty of Advanced Life Science, Hokkaido University, Sapporo, 060-0810 Japan; 2 Department of Chemistry and Applied Chemistry, Faculty of Science and Engineering, Saga University, Saga, 840-8502 Japan; 3 Global Station for Soft Matter, Global Institution for Collaborative Research and Education, Hokkaido University, Sapporo, 001-0021 Japan; 4 Department of Life Science and Institute of Biological Interfaces, Sogang University, Seoul, 04107 Republic of Korea

**Keywords:** Molecular biophysics, Permeation and transport

## Abstract

Many microbial rhodopsins self-oligomerize, but the functional consequences of oligomerization have not been well clarified. We examined the effects of oligomerization of a H^+^ pump, *Gloeobacter* rhodopsin (GR), by using nanodisc containing trimeric and monomeric GR. The monomerization did not appear to affect the unphotolyzed GR. However, we found a significant impact on the photoreaction: The monomeric GR showed faint M intermediate formation and negligible H^+^ transfer reactions. These changes reflected the elevated pKa of the Asp121 residue, whose deprotonation is a prerequisite for the functional photoreaction. Here, we focused on His87, which is a neighboring residue of Asp121 and conserved among eubacterial H^+^ pumps but replaced by Met in an archaeal H^+^ pump. We found that the H87M mutation removes the “monomerization effects”: Even in the monomeric state, H87M contained the deprotonated Asp121 and showed both M formation and distinct H^+^ transfer reactions. Thus, for wild-type GR, monomerization probably strengthens the Asp121-His87 interaction and thereby elevates the pKa of Asp121 residue. This strong interaction might occur due to the loosened protein structure and/or the disruption of the interprotomer interaction of His87. Thus, the trimeric assembly of GR enables light-induced H^+^ transfer reactions through adjusting the positions of key residues.

## Introduction

The cell membrane is a very thin film compared to the cell diameter but has a substantial capacity to execute various reactions, such as energy conversions, signal transductions, and the capture and expulsion of substances. These vital functions are mainly fulfilled by the proteins in the membrane. Microbial rhodopsin is the most abundant membrane protein in the microbial world^1,2^. Like animal rhodopsins, it contains the chromophore retinal in the central pocket surrounded by seven transmembrane helices. Upon light illumination, the retinal undergoes isomerization, which in turn distorts the protein conformation. This energized state is thermally relaxed to the original state through several structural intermediates. During this cyclic reaction, called the photocycle, microbial rhodopsin performs various roles, including light-signal transductions, light-driven ion pumps, light-gated ion channels, and even light-switchable enzymes. Consequently, microbial rhodopsins enable the cellular utilization of light not only as a signal source for phototaxis behaviors and chromatic adaptations but also as an energy source for ATP production and other metabolic processes.

In cell membranes, microbial rhodopsins are known to adopt oligomeric states. This association is essential for archaeal sensory rhodopsins (SRs), which mediate cellular phototaxis behaviors^1,2^. Upon light absorption, SRs transmit light signals to the cognate transducers embedded in the membrane. Thus, SRs and transducers need to form heterodimers even in the dark state. On the other hand, most other microbial rhodopsins adopt homooligomeric states: Ion pumps and eubacterial sensors form complexes with 3–6 units^3–11^, while ion channels form homodimers^12–15^. The monomers appear to be equipped with all machineries for their respective functions. Thus, the functional consequences of oligomer formation have not been fully understood.

The archaeal H^+^ pump bacteriorhodopsin (BR) is the best studied rhodopsin in the microbial world^16^. Native BR forms a trimer that is further organized into a two-dimensional hexagonal lattice^3^. Compared to this native state, monomeric BR has weaker structural stability and a 30% lower isomeric composition of all-*trans* retinal^17,18^. Most microbial rhodopsins are functional only in the “all-*trans*” form. Thus, the oligomeric state of BR is advantageous for higher activity but is not essential for the function itself^19^. Monomerization effects were also examined for the eubacterial H^+^ pump proteorhodopsin (PR), and both advantages and disadvantages were reported^20,21^. The monomeric PR shows a faster photocycle, which means a faster turnover rate and thus elevates the total amount of transported H^+^. On the other hand, monomerization elevates the pKa of Asp97, which acts as the “H^+^ acceptor” for the protonated Schiff base. This primary H^+^ transfer is a prerequisite for pump activity and occurs only when the H^+^ acceptor is deprotonated in the dark state. Thus, higher pKa narrows the pH range where the H^+^ pump exerts the activity. The observed pKa increase was approximately 1.1 unit^21^. The resultant pKa of 7.5–7.7 is still equal to or lower than the seawater pH of 7.6–8.2^22^. Thus, even in the monomeric state, PR can pump H^+^ in the physiological environment. In contrast to BR and PR, oligomer formation appears essential for the archaeal Cl^−^ pump halorhodopsin (HR). Upon monomerization, HR shows a greatly distorted photocycle, implying significantly lowered activity^23^. However, the monomeric state was achieved by amino acid replacement, which might also distort the photocycle. Thus, the “net” monomerization effect is not fully understood for HR.

**Figure 1:**
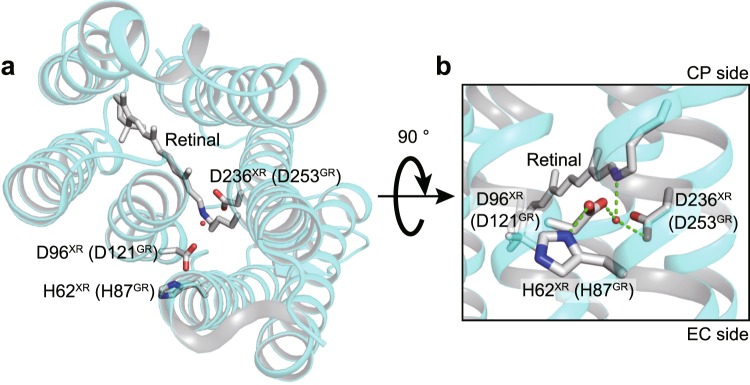
The positions of key residues in this study. Here, we showed the structure of XR (PDB ID: 3DDL), where the “H^+^ acceptor” Asp96 residue interacts with the neighboring His62 residue. They correspond to Asp121 and His87 in GR, respectively. The top view from the cytoplasmic (CP) side is shown in the left panel. Enlarged view of the H^+^ acceptor region is shown in the right panel. Here, “EC side” means “extracellular side”. The broken lines represent the proposed hydrogen bonds, and the red sphere represents water molecule.

In this study, we examined the effects of monomerization on the H^+^ pump *Gloeobacter* rhodopsin (GR) from the cyanobacterium *Gloeobacter violaceus*^24,25^. GR is a representative member of the xanthorhodopsin (XR) family, which is one of three major families of bacterial H^+^ pumps. The other two are the BR and PR families described above (for review, see^26^). BR and its homologs are found in extremely halophilic archaea. On the other hand, eubacterial H^+^ pumps are widespread in cells inhabiting a broad range of environments, and most of them are phylogenetically classified into either PR or XR families^26^. PR is the first eubacterial rhodopsin discovered through the metagenomic analysis of marine samples^27^. The first PR gene was identified in the DNA fragment from gamma-proteobacteria. Later, PR-like genes were revealed to be encoded in various oceanic eubacteria (for review, see^28^). Compared to the PR family, the H^+^ pumps of the XR family are relatively abundant in nonmarine environments. XR is the first characterized member, which was obtained from the eubacterium *Salinibacter ruber* inhabiting a salt pond^29^. GR is closely related to XR (~70% amino acid similarity), but its host strain was isolated from calcareous rock^24^. Moreover, many members have also been identified in fresh and brackish water environments^30,31^. Despite forming distinct phylogenetic clusters, PR-like and XR-like proteins share many residues. One notable example is a histidine (His75 for PR, His62 for XR, His87 for GR) that forms a salt bridge with the H^+^ acceptor Asp residue^9,32,33^ (Fig. 1). This His residue is replaced by Met in BR (Fig. S1) and thus is characteristic only of eubacterial H^+^ pumps. For PR, the His-Asp interaction was shown to elevate the pKa of the Asp residue^33^, and the His residue is believed to participate in the H^+^ relay reactions during the photocycle^34^. One prominent difference between PR and GR is the accommodation of carotenoid antenna: XR-like proteins can accommodate a keto-carotenoid, which is the second chromophore to harvest light energy^29^. The residues for carotenoid binding are conserved only in XR-like proteins, but this binding is not a prerequisite for the H^+^ pumping ability. Indeed, heterologously expressed GR in *Escherichia coli* and *Xenopus* oocytes lacked carotenoid binding but showed strong H^+^ pumping activity^35,36^. Moreover, some host cells of XR-like proteins lack the genes for keto-carotenoid synthesis^37,38^.

Previously, we reported that detergent-solubilized GR shows a pH-dependent multiplicity of oligomeric states^39^: At neutral and alkaline pH, GR forms trimers and higher oligomers. However, this assembly disrupts into monomers upon acidification, probably reflecting the protonation of Asp121, which is the H^+^ acceptor for GR. At a weakly acidic pH, an equilibrium between the monomer and trimer is formed. Thus, in this study, we trapped these forms in nanodiscs and used them for comparative studies. The experimental results indicated the crucial importance of oligomerization for the H^+^-pumping function.

## Results

### Nanodiscs containing monomeric and trimeric GR

**Figure 2:**
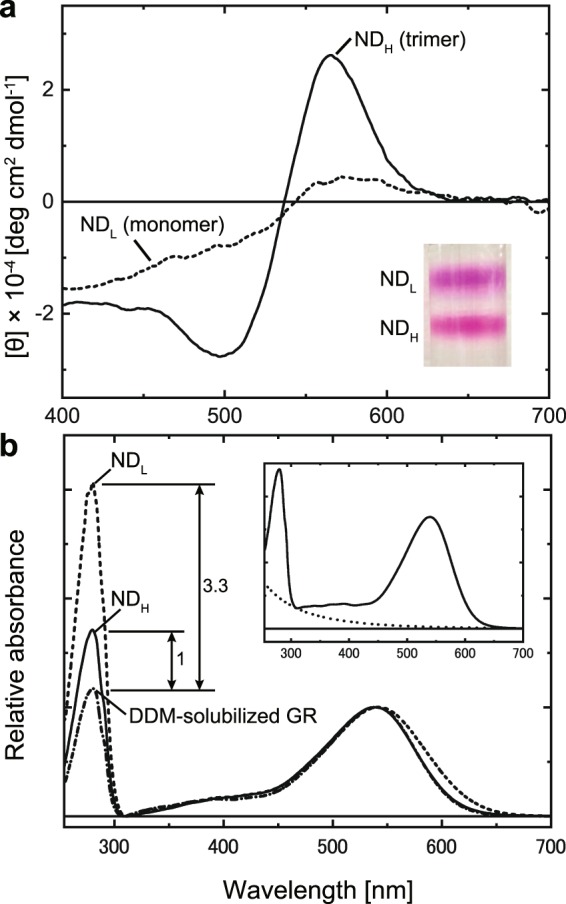
Comparison of CD (**a**) and absorption (**b**) spectra between two nanodiscs. The inset of panel a shows the nanodisc bands isolated by sucrose density gradient centrifugation. In panel b, the spectrum of DDM-solubilized GR is also shown. Here, all three spectra were plotted after subtracting the scattering contribution as shown in the inset, which shows the raw spectrum of the DDM-solubilized state and the estimated scattering artifact (for details, see Materials and Methods section). The buffer solution was 6-mix buffer, pH 7, containing 0.3 M NaCl. For DDM-solubilized GR, the medium was supplemented with 0.05% DDM.

Previously, we examined the oligomeric state of GR solubilized by the detergent n-dodecyl-β-D-maltoside (DDM)^39^. In size exclusion chromatography, three peaks appeared with changing magnitudes depending on pH. Based on the theoretical mass calculations, we assigned them as a monomer, trimer, and higher oligomer of GR. At alkaline pH, GR is in either trimeric or higher oligomeric states. At approximately pH 7, trimeric GR becomes dominant. Upon further acidification, GR disrupts into monomers, which become dominant below pH 4. This trimer-to-monomer transition occurs almost simultaneously with the protonation of the Asp121 residue. Moreover, when the adjacent His87 residue is replaced with Met, the monomeric state becomes dominant at any pH. Thus, the oligomeric state seems highly sensitive to the His-Asp interaction, though the mechanism is still unclear. Here, we utilized this pH-dependent behavior to prepare monomeric and trimeric GR. At a weakly acidic pH, the amounts of GR in these two states became almost equal. Thus, at pH 5.5, we reconstituted them into nanodiscs. The resultant mixture of nanodiscs probably containing monomeric and trimeric GR was then separated by sucrose density gradient centrifugation. The inset of Fig. 2a shows the centrifugal tube, in which two distinct bands of GR nanodiscs were observed. These bands were collected separately and used for the subsequent studies. Hereafter, we refer to the nanodiscs at higher and lower densities as ND_H_ and ND_L_, respectively. Figure 2a shows their circular dichroism (CD) spectra, which are quite similar to those for trimeric and monomeric GR in the DDM-solubilized state^39^. When microbial rhodopsin is in the oligomeric state, biphasic bands appear in the visible region in many cases^40,41^. These bands reflect the exciton coupling among retinals in adjacent rhodopsins and thus indicate oligomer formation. Such a CD spectrum was observed for ND_H_, where clear negative and positive bands appeared at approximately 500 and 570 nm, respectively. In contrast, for ND_L_, only a faint band appeared at approximately 570 nm. This pattern indicates that GR is in the monomeric state. Figure 2b shows the absorption spectra of ND_L_, ND_H_, and GR in the DDM-solubilized state at pH 7. To evaluate the peaks at approximately 280 nm, the spectra were plotted after the removal of scattering artifacts, which were estimated as shown in the inset (for details, see Materials and Methods section). The resultant spectra indicate that the band shapes at approximately 540 nm are almost the same, and the only differences appear in the magnitudes of the 280 nm band. Two nanodiscs have larger absorptions due to the contributions of membrane scaffold protein (MSP). Here, we calculated the absorption differences (ΔA_280_) from the peak of DDM-solubilized GR: The value for ND_L_ is 3.3-fold larger than that for ND_H_. In this figure, three spectra were plotted so that the peak magnitudes at approximately 540 nm become identical. Thus, the “difference in ΔA_280_” indicates the “difference in MSP amount”, making it possible to incorporate the same amount of GR into respective nanodiscs. The value “3.3-fold” means that the MSP amount is 3.3-fold larger for ND_L_ than for ND_H_. This result supports our expectation that ND_H_ and ND_L_ incorporate trimeric and monomeric GR, respectively.

In the DDM-solubilized state, the GR trimer disrupts into monomers upon acidification^39^. This pH dependence was examined for trimeric GR in nanodiscs. Figure S2 shows the CD spectra at pH 7 and 4 of DDM-solubilized GR (panel **a**) and the ND_H_ (nanodisc containing GR trimer) (panel **b**). Here, a clear contrast appeared. For the DDM-solubilized state, the biphasic band disappeared at pH 4. However, for the nanodisc, the biphasic band remained even at pH 4. Thus, acid-induced monomerization does not appear to occur for the GR trimer within the lipid membrane.

### Monomerization effects on the photochemical characteristics

#### Photocycles

**Figure 3:**
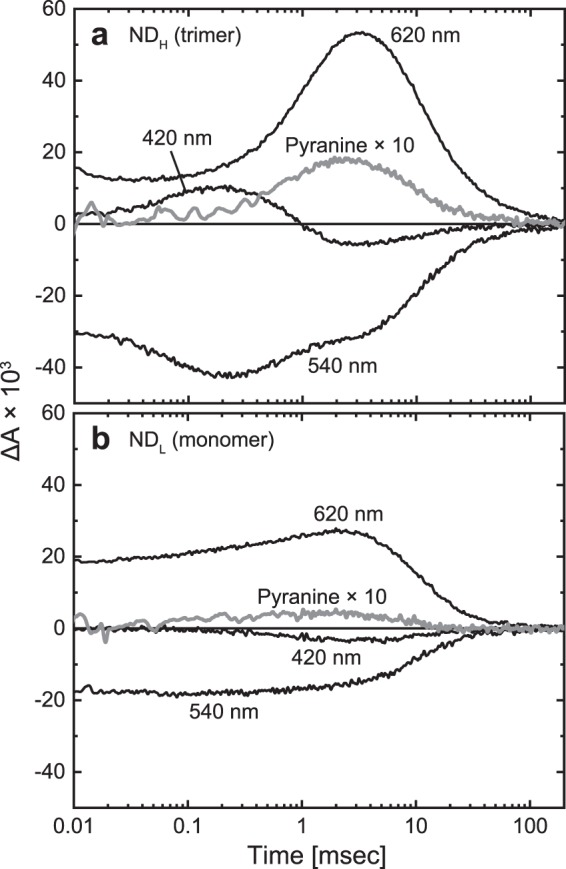
Comparison of photocycles between trimeric and monomeric GR. Flash-induced absorbance changes at typical three wavelengths were plotted for the GR trimer in ND_H_ (**a**) and the monomer in ND_L_ (**b**). The buffer solution was 6-mix buffer, pH 7, containing 0.3 M NaCl. The transient pH changes were detected by 100 μM pyranine, whose absorbance changes at 457 nm were measured in 0.5 mM MOPS (pH 7) containing 0.3 M NaCl and plotted here at 10-fold magnification. All samples contained 10 μM GR.

As shown in Fig. 2b, monomeric and trimeric GR have almost the same “retinal spectrum”. There are no significant differences in peak positions and band widths. However, we found significant differences in the photocycles. Figure 3 shows the time-dependent absorbance changes at pH 7 after flash excitation. Data for both trimeric and monomeric GR were measured in samples containing the same concentration of GR. The trimeric GR (panel **a**) shows a “normal” photoreaction, which is essentially the same as that observed within the lipid membrane at neutral pH^25^. In the early time range (~0.01 msec), K and subsequent L intermediates are in equilibrium: They appear at 620 and 540 nm, respectively. With concomitant decay, the M intermediate appears at 420 nm and reaches maximum accumulation at approximately 0.2 msec. The subsequent decay of M leads to the formation of O at 620 nm through a faint accumulation of N at 540 nm at approximately 1 msec. During the O decay, GR returns to the original dark state. On the other hand, the monomeric GR (panel **b**) shows a significantly distorted photocycle, where K-like intermediate also appears in the early time range, but other intermediates are almost absent. Thus, most K-like intermediates seem to directly return to the original state. It is likely that a small amount of M also exists in the early time range and then decays at approximately 1 msec. However, its signal is very faint, and thus, the signal of the subsequent O formation is also faint. Reflecting the slight accumulation of O, only a slight increase was observed at 620 nm at approximately 1 msec.

The M formation indicates the primary H^+^ transfer from the Schiff base to the H^+^ acceptor Asp121 residue and is a prerequisite for the subsequent H^+^ transfer reactions: (1) The reprotonation of the Schiff base by the H^+^ from Glu132 in the cytoplasmic (CP) channel, (2) the uptake of H^+^ by Glu132 from the CP medium, and (3) the release of H^+^ from Asp121 to the extracellular medium. These reactions sequentially occur at the M-to-N, N-to-O, and O-to-original state transitions, respectively. To examine the H^+^ transfer reactions, we used the pH indicator dye pyranine, which can detect the transient pH changes due to reactions (2) and (3) described above. In Fig. 3, the absorption change of pyranine at 457 nm is also plotted after 10-fold expansion. For trimeric GR, a distinct absorption change was observed during the formation and decay of O. The positive shift indicates transient alkalization due to reaction (2). Subsequently, the pyranine signal returned to the baseline reflecting the reaction (3). Thus, the trimeric GR undergoes “normal” H^+^ transfer reactions, where the H^+^ uptake precedes the H^+^ release. For monomeric GR, a pyranine signal associated with O accumulation was also observed. However, it was very faint, reflecting the slight accumulation of O and its precursor M. Thus, the monomeric GR probably has negligible H^+^-pump activity.

#### Retinal configurations

**Figure 4:**
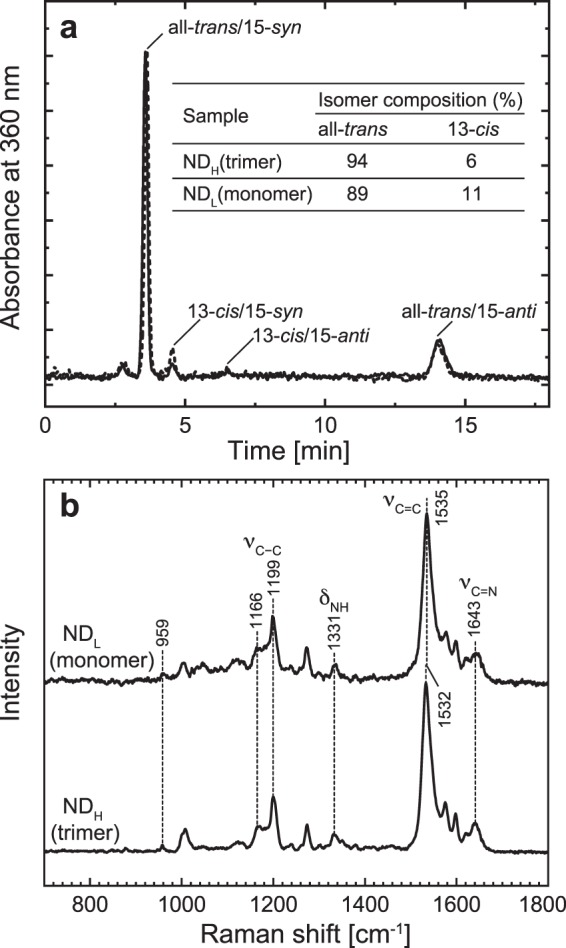
HPLC analysis and resonance Raman spectra. Panel a shows the HPLC chromatographs of retinal isomers extracted from the dark-adapted GR in ND_H_ (solid line) and ND_L_ (broken line). The nanodiscs were suspended in 6-mix buffer, pH 7, containing 0.3 M NaCl. The ratios of all-*trans* and 13-*cis* retinals were determined from the peak areas and are indicated in the panel. Panel b shows the resonance Raman spectra of unphotolyzed GR with 441.6 nm excitation. The nanodiscs were suspended in 20 mM MOPS, pH 7, containing 0.3 M NaCl.

Unphotolyzed BR can accommodate both all-*trans* and 13-*cis* retinal and shows so-called “light-dark adaptation”^1^. In the light-adapted state after continuous illumination, the unphotolyzed BR predominantly contains all-*trans* retinal. Upon dark adaptation, the 13-*cis* content increases up to 50%. As mentioned above, the monomerization of BR affects the retinal isomer composition: Even in the light-adapted state, the 13-*cis* content becomes approximately 30%^17^. The 13-*cis* BR is known to result in the photocycle lacking the M intermediate^42^. Thus, we examined the isomer compositions of trimeric and monomeric GR. In contrast to BR, GR within the membrane was reported to lack light-dark adaptation and always maintain predominantly all-*trans* retinal^25^. Figure 4a shows the HPLC chromatographs of the extracted retinal. As shown here, both trimeric and monomeric GR predominantly contain all-*trans* retinal. These retinals were extracted after GR was kept in a dark environment (dark-adapted state), but almost the same results were obtained for light-adapted GR (data not shown). The same conclusion was also drawn from the resonance Raman spectra in Fig. 4b. These spectra were measured with 441.6 nm excitation and originated from the retinals in the unphotolyzed states. As shown here, the spectra of trimeric and monomeric GR are essentially the same, and they are almost the same as the spectrum in the DDM-solubilized state^43^. Here, most bands appear at the same positions with the same intensities. The prominent bands at approximately 1535 cm^−1^ are the ethylenic stretch (ν_C=C_) vibrations. For retinal compounds, there exists an approximately linear correlation between the absorption maximum wavelength (λ_max_) and the ethylenic stretching frequency (ν_C=C_). The 1535 cm^−1^ frequency correlates with the λ_max_ of 537 nm^43–45^, which is close to the measured values of 540 nm for trimeric GR and 543 nm for monomeric GR. These λ_max_ values in the visible region reflect the protonations of the Schiff bases, which are consistent with the Raman bands at 1643 and 1331 cm^−1^. They are assigned to the C=N stretching (ν_C=N_) and N-H bending (δ_N-H_) modes of the Schiff base linkage, respectively. These bands should shift or disappear in response to a change in the hydrogen-bonding environment or the deprotonation of the Schiff base. Thus, their appearances at almost the same positions reflect that both Schiff bases are protonated and surrounded by similar environments. For retinal, the C-C skeletal stretching modes in the 1100–1400 cm^−1^ region are called the “fingerprint region” and are sensitive to the retinal configuration^46^. The regions of both trimeric and monomeric GR commonly consist of bands at 1199 and 1166 cm^−1^. These bands are indicative of the all-*trans* configuration. The Raman spectrum is highly sensitive to the deformations of the retinal polyene chain, which is observed by relatively strong hydrogen-out-of-plane (HOOP) wagging vibrations in the low-frequency region. Both spectra show only small HOOP bands at only 959 cm^−1^ with almost the same intensity, reflecting small and similar distortions of both retinals.

#### pKa of the Asp121 residues

**Figure 5:**
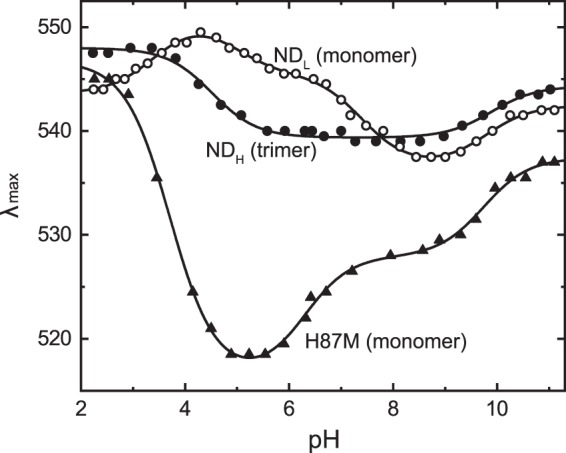
The pH-dependent shifts of absorption λ_max_. The λ_max_ values at various pH values were examined for three nanodiscs containing wild-type trimer, wild-type monomer, and the monomer of the H87M mutant. The smooth lines are the best-fitted results with the linear combinations of Henderson-Hasselbalch functions. The determined pKa values were 4.52 and 9.89 for ND_H_, 3.43, 4.95, 7.41 and 9.78 for ND_L_, and 3.72, 6.30 and 9.74 for H87M. The medium contained 6-mix buffer and 0.3 M NaCl. The pH was adjusted by adding HCl or NaOH.

For the primary H^+^ transfer reaction, the H^+^ acceptor needs to be deprotonated in the dark state. Thus, we examined the pKa of this residue by pH-induced spectral shifts. Figure 5 shows the plots of the absorption λ_max_ values against pH. Here, the best fitted results with the sum of Henderson-Hasselbalch functions are also plotted with smooth lines. As shown here, both monomeric and trimeric GR show complicated spectral shifts, probably reflecting changes in the protonation states of several residues, whereas the protonation of the H^+^ acceptor should cause a relatively large redshift upon acidification. Such a shift occurs at pH 5.5 → 4 for trimeric GR and pH 8.5 → 4.5 for monomeric GR. Thus, monomerization significantly shifts the “redshift region” toward higher pH. The determined pKa for the redshift is 4.52 for trimeric GR. For monomeric GR, two pKa values were needed: The values are 4.95 and 7.41. This elevated pKa probably increases the fraction of protonated Asp121 at pH 7 and then induces limited H^+^ transfer reactions.

**Figure 6:**
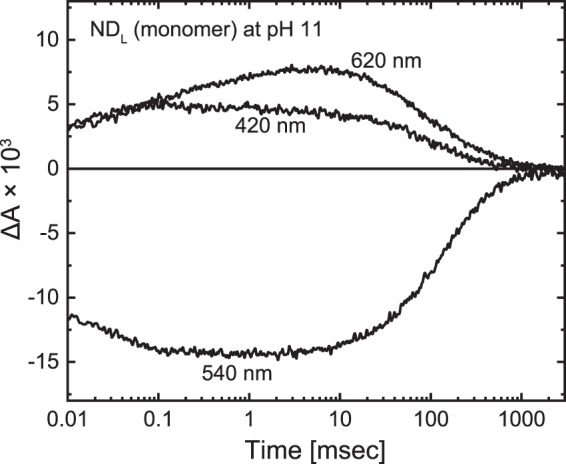
Photocycle of monomeric GR at pH 11. The experimental conditions were the same as in Fig. 3b except for the pH.

Even for monomeric GR, the Asp121 residue should deprotonate at higher pH, and then M accumulation should become prominent. Figure 6 shows the flash-induced absorbance changes of monomeric GR at pH 11. As expected, the M accumulation became significant. Its decay occurred at approximately 100 msec, which was approximately 100-fold slower than that for trimeric GR at pH 7 (Fig. 3a). This result probably reflects the malfunction of the Glu132 residue in the CP channel. At such an alkaline pH, this residue might not act as an efficient H^+^ donor for the deprotonated Schiff base.

### H87M mutant

**Figure 7:**
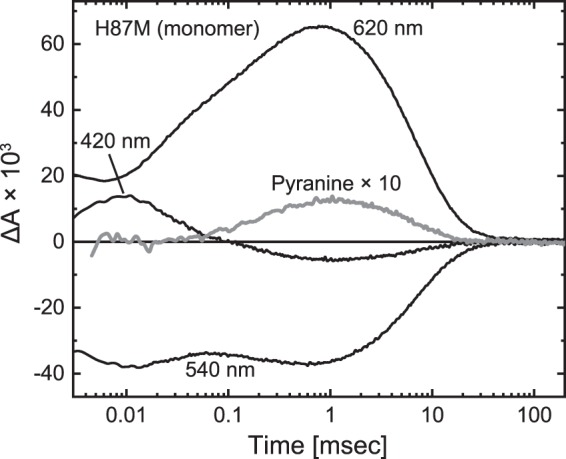
Photocycle of monomeric H87M mutant in nanodisc. The experimental conditions were the same as in Fig. 3.

The significant pKa shift should reflect an altered environment around the Asp121 residue upon monomerization. Here, we focused on His87 of GR, which is located close to Asp121. As mentioned above, H87M GR was monomeric in the DDM-solubilized state at any pH^39^. Despite the monomeric state, this mutant had a pKa of approximately 4, which is significantly lower than the pKa values of 4.95 and 7.41 for the wild-type monomer in the nanodisc (Fig. 5). These results suggest that upon monomerization, the His87 residue might significantly elevate the pKa. The pKa value for H87M described above was determined in the DDM-solubilized state^39^. Thus, we made nanodiscs containing H87M GR. Reconstitution was performed at pH 7 (not at pH 5.5), and the resultant nanodisc contained the monomer of H87M, which was confirmed by sucrose density gradient centrifugation (Fig. S3). Upon centrifugation, the nanodisc made a single band, whose position was identical to that of ND_L_ containing the wild-type monomer. Despite this monomeric state, H87M undergoes the photocycle containing the M intermediate, as shown in Fig. 7. This mutant showed a relatively faster photoreaction, where M and O clearly appeared as in the photocycle of the wild-type trimer (Fig. 3a). Reflecting their accumulations, the pyranine signal was also significant. Thus, H87M appears to have the H^+^ pump ability even in the monomeric state. We also examined the pKa of Asp121 for H87M GR. The results are shown in Fig. 5. Similar to the DDM-solubilized state^39^, this mutant has a low pKa of approximately 3.72 in the nanodisc and thus has a deprotonated Asp121 at pH 7. For wild-type GR, the His87-Asp121 interaction probably becomes stronger upon monomerization and then elevates the pKa of Asp121.

The mutation-induced acceleration of the photoreaction was also observed in the corresponding mutant of PR such as H75N^33^. These observations might reflect the tautomerization and conformational changes of His sidechains upon M formations. These dynamic changes were recently revealed for PR by solid-state NMR analyses^47^.

### Sensitivities to hydroxylamine and heat treatment

**Figure 8:**
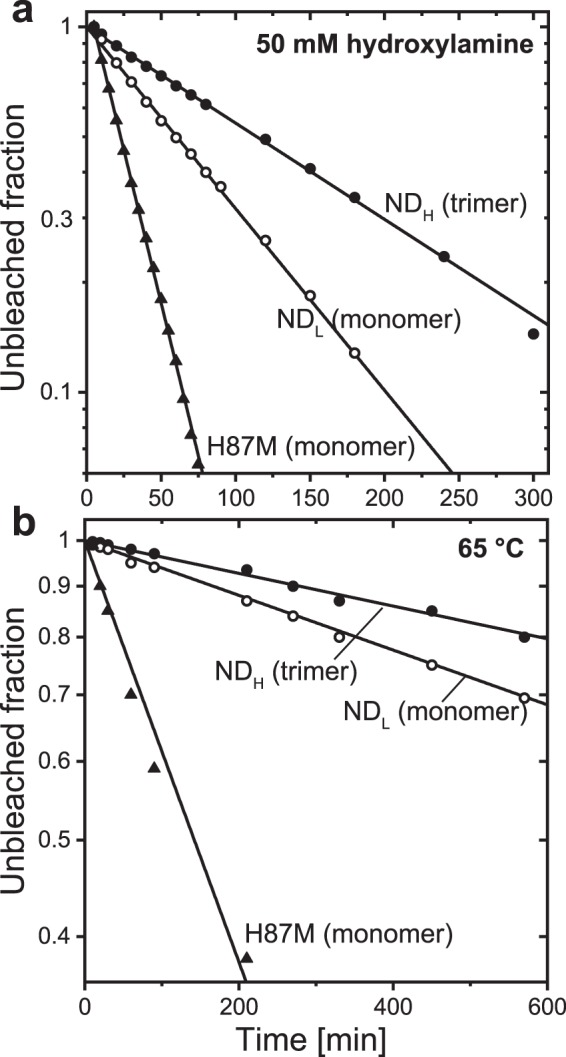
Sensitivities to hydroxylamine and heat treatment. The bleaching kinetics due to 50 mM hydroxylamine (**a**) and thermal denaturation (**b**) were examined. Both panels show the remaining amounts of GR, which were evaluated from the absorbance values at the respective absorption λ_max_. The bleaching rates were determined by fitting with a single exponential function. The determined rates are as follows: For panel a, 6.07 × 10^−3^ min^−1^ for ND_H_, 1.15 × 10^−2^ min^−1^ for ND_L_, and 3.85 × 10^−2^ min^−1^ for H87M; for panel b, 3.79 × 10^−4^ min^−1^ for ND_H_, 6.29 × 10^−4^ min^−1^ for ND_L_, and 4.88 × 10^−3^ min^−1^ for H87M. Both experiments were performed in the dark. The nanodiscs were incubated in pH 7 buffer (20 mM MOPS, 0.3 M NaCl) at 25 °C for panel a and pH 8.5 buffer (50 mM HEPES, 0.3 M NaCl) at 65 °C for panel b.

Upon monomerization, the lateral pressure on GR probably becomes weak. This change might loosen the protein structure. To probe this possible monomerization effect, we examined the sensitivities to hydroxylamine and heat treatment. The results are shown in Fig. 8a,b, respectively. If hydroxylamine can diffuse into the protein interior, it breaks the Schiff base linkage. The resultant retinal oxime has absorption at approximately 360 nm, and thus, the absorption at λ_max_ at approximately 540 nm decreases (panel **a**). A similar change in the absorption spectrum also occurs due to thermal denaturation (panel **b**). High temperature distorts the protein conformation. Then, the Schiff base tends to be exposed to the medium and thus broken. In both panels, the relative absorption values at λ_max_ are plotted against the incubation time. At room temperature (panel **a**), bleaching did not occur in the absence of hydroxylamine. In the presence of 50 mM hydroxylamine, bleaching occurred in both trimeric and monomeric GR. The bleaching rate of the monomer (1.15 × 10^−2^ min^−1^) was 1.9-fold faster than that of the trimer (6.07 × 10^−3^ min^−1^). The faster bleaching of monomeric GR also occurred at 65 °C incubation (panel **b**), where the bleaching rate of the monomer (6.29 × 10^−4^ min^−1^) was 1.7-fold faster than that of the trimer (3.79 × 10^−4^ min^−1^). Thus, the monomerization actually loosened the structure.

Figure 8 also shows the data for H87M GR. This mutant showed even faster bleaching than the wild-type monomer in both experiments. Thus, this mutation further distorts the protein structure. The effect of this mutation and the role of residue His87 will be discussed later.

## Discussion

### GR oligomer in nanodisc

In this study, we examined the functional consequences of oligomer formation for GR. Here, we reconstituted GR into nanodiscs. From our previous experiments^39^, we expected to obtain two kinds of nanodiscs, containing monomeric and trimeric GR. This prospect was supported by the absorbance differences at 280 nm in Fig. 2b. In contrast, recent high-speed AFM imaging showed a pentameric state of GR within the nanodisc^11^. This discrepancy might originate from the differences in pH and/or lipid: we made nanodiscs at pH 5.5 with 1-palmitoyl-2-oleoylphosphatidylcholine (POPC), while the AFM study adopted pH 7.4 and asolectin, respectively. As mentioned above, the solubilized GR tends to form higher oligomers at alkaline pH. At approximately pH 7.4, there exists a trimer and a higher oligomer of GR^39^. The higher oligomer might be preferentially observed by AFM, revealing its pentameric state. As shown in Fig. 2a, the trimeric GR shows a biphasic CD spectrum. Similar spectra were also measured for GR in the *E. coli* membrane^39^ and in the pentameric state within the nanodisc^11^. Thus, GR should exhibit an oligomeric state in the cell membrane, but the number of monomers and the effect of keto-carotenoid binding should be clarified in future investigations. In the following sections, we discuss the functional consequences of trimer formation.

### The pKa of the Asp121 residue

The monomerization of GR did not influence the absorption spectrum or the retinal configuration but significantly elevated the pKa of the Asp121 residue. The H^+^ pump function was thus strongly hampered even at neutral pH. For monomeric GR, the acid-induced redshift of λ_max_ was described by two pKa values of 4.95 and 7.41. Thus, another acidic residue contributes (residue X). At pH 7, we observed only negligible M accumulation and H^+^ transfer reactions, showing that most GR contains the protonated Asp121 residue. Thus, the higher pKa (7.41) most likely represents the protonation state of the Asp121 residue. For the wild-type trimer and H87M mutant, the acid-induced redshifts were described by only a single pKa, indicating a negligible influence of residue X. Thus, the deprotonation of residue X becomes measurable upon monomerization but is not measurable even in the monomeric state when His87 residue is absent. Residue X seems to be located near either His87 or Asp121, and its pKa might increase when the His-Asp interaction becomes strong due to monomerization. One possible candidate might be Asp253, which is superconserved residue in microbial rhodopsin and probably makes hydrogen-bonding network with Asp121, Schiff base, and one water molecule. The corresponding networks in XR and BR are shown in Figs 1 and S1, respectively. Similar to other H^+^ pumps, the pKa of Asp253 is probably very low in the wild-type trimer and H87M mutant, and thus this residue might be deprotonated over the entire pH range investigated. This pKa might be elevated to 4.95 in the wild-type monomer, and so the deprotonation might become detectable. However, unequivocal assignment of the residue X must await further study.

### The pKa increase via stronger His-Asp interaction

The His87 of GR is a superconserved residue among the XR and PR families. Their His residues are known to interact with the H^+^ acceptor Asp residues^9,32,33^. For PR, this interaction was reported to elevate the H^+^ acceptor pKa (Asp97) by approximately 0.8–2.2 pH units^33^, although the mechanism is still unclear. Here, we found that the His-Asp interaction of GR becomes stronger upon monomerization and significantly increases the pKa of the Asp121 residue, which is fatal for the H^+^ pump function. As mentioned above, a relatively small but similar pKa increase was also observed for PR upon monomerization^20,21^. Thus, the stronger His-Asp interaction might occur for both XR and PR. On the other hand, monomeric BR does not have an elevated pKa despite the weakened structural stability similar to monomeric GR^18^. This result might reflect the lack of conservation of the His residue, which is replaced by Met56 in BR.

Why does the His-Asp interaction become stronger upon monomerization? As shown in Fig. 8, the monomeric GR adopts a loosened structure, which might allow the His87 residue to adopt a preferable position for stronger His-Asp interaction. An additional possibility might be another interaction of the His87 residue in the trimeric state. This kind of interaction was observed in the solid-state NMR spectra of PR^47^ and the crystal structures of blue-light-absorbing PRs (BPRs)^9^, which are closed homologs of PR. Their structures show hexameric or pentameric rings of BPRs, where the His residues (H75) are located near the oligomer interfaces and form hydrogen bonds with Trp34 residues from the neighboring BPRs (Fig. S4). Thus, each His residue forms the “intraprotomer” His-Asp interaction and the “interprotomer” His-Trp interaction. Monomerization disrupts the His-Trp interaction, which might strengthen the remaining His-Asp interaction. In this study, we prepared trimeric GR. Even in this trimeric state, the His residue might interact with a residue from the neighboring GR. The Trp34 of PR corresponds to Ser46 in GR, which can form a hydrogen bond with the His residue. However, the interacting partner might not be the Ser46 residue due to the different GR arrangements in the hexameric and pentameric states. In the DDM-solubilized state, GR cannot form trimers at any pH when the His87 residue is absent^39^. This observation might reflect the contribution of the His87 residue to stabilizing the trimeric assembly through the “interprotomer” interaction.

### The functional consequences of trimer formation

The monomeric GR adopts a loosened structure, as determined by the sensitivities to hydroxyl amine and heat treatment. These high sensitivities reflect the hydrophilic environment of the Schiff base region, even in the dark state. Upon illumination, GR should undergo further conformational change, and thus, the loosened structure in the dark state should increase the risk that the Schiff base linkage might be broken during the photocycle. Thus, the trimer formation of GR has two advantages. First, trimer formation leads the proteins to have moderate rigidity, which allows the structural changes that enable H^+^ pump function but prevents denaturation during the photocycle. Second, in the trimeric form, the His87-Asp121 interaction becomes weaker due to the trimerization-induced conformational change and/or the formation of an interprotomer interaction of the His87 residue. The resultant low pKa of Asp121 is a prerequisite for primary H^+^ transfer upon photoexcitation.

Even in the monomeric state, GR can perform the H^+^ pump function if the His87 residue is absent. Thus, for a GR ancestor, there seemed to be two choices to attain H^+^ pump function: One is to adopt the monomeric state without conserving the His87 residue, and the other is to take the trimeric state while conserving the His87 residue. The former is probably high in risk: The H87M mutant has a looser than the wild-type monomer (Fig. 8); thus, this mutant most likely shows low tolerance for repeated photoexcitations. Thus, trimer formation is the rational choice for GR to acquire both structural stability and H^+^ pump ability.

## Materials and Methods

### Protein expression and purification

The construction of expression plasmids for C-terminal His-tagged wild-type GR and the H87M mutant was reported previously^39^. The proteins were prepared as described^48^. Briefly, these proteins were expressed in *E. coli* BL21(DE3) cells. After solubilization with DDM, they were purified by a Ni-NTA agarose column. GR concentrations were estimated by supposing the molar extinction coefficient at λ_max_ to be 50,000 M^−1^ cm^−1 ^^49^.

The MSP, MSP1E3D1, was prepared according to standard protocols^50^. Briefly, the pET28a plasmid containing the MSP1E3D1 gene (Addgene plasmid #20066) was transformed into *E. coli* BL21(DE3) cells, which were then grown at 37 °C in TB medium supplemented with 50 μg/mL kanamycin. At the late exponential growth phase, expression was induced by the addition of 1 mM isopropyl-β-D-thiogalactopyranoside. After 1 h, the medium was cooled to 28 °C, and induction was continued. After 3.5–4 h, the cells were harvested by centrifugation (6,400 × g, 8 min at 4 °C). The expressed MSP has an N-terminal 7 × His tag and thus was purified by a Ni-NTA agarose column after cell disruption by a French press. The fractions containing MSP were mixed, and the buffer solution was replaced with 5 mM Tris-HCl, pH 7, containing 50 mM NaCl and 0.05 mM EDTA by ultrafiltration. The resultant MSP solution was stored at −30 °C. The MSP concentration was calculated by using ε_280_ = 29,900 M^−1^ cm^−1^.

### Assembly and separation of nanodiscs

The nanodiscs containing GR were prepared according to standard protocols with some modifications^50^. Initially, the buffer solution of GR was replaced by dialysis against 3-mix buffer (27.4 mM citric acid, 9.2 mM ADA, 20.8 mM MOPS), pH 7, containing 0.3 M NaCl and 0.1% DDM. For wild-type GR, the buffer pH was adjusted to 5.5 with HCl before mixing with MSP and lipid. At this pH, the DDM-solubilized GR takes equilibrium between the trimeric and monomeric states^39^. The H87M GR takes a monomeric state at any pH^39^, and thus, the pH was not changed from 7. These GR solutions were mixed with the MSP solution described above and the lipid solution (10 mg/mL in 25 mM cholic acid). Here, we used POPC for the lipid. The initial pH values of 5.5 and 7 were maintained throughout the procedures. The resultant mixtures contain three components (GR, MSP, POPC) with molar ratios of 3: 2: 200 for wild-type GR and 1: 2: 200 for the H87M mutant. After 20 min of incubation on ice, the detergents were removed at 4 °C for 12 hours by the addition of ~500 mg SM2 Adsorbent Bio-Beads (Bio-Rad, Hercules, CA, USA) per mL of solution.

The nanodiscs containing monomeric and trimeric GR were separated by linear 10–40% (w/v) sucrose density gradient centrifugation at 40,000 rpm in SW41Ti rotor (Beckman) for 16 h at 4 °C. The bands of nanodiscs were collected separately, and the sucrose was replaced with appropriate buffers by ultrafiltration. For several experiments, the nanodiscs were suspended in 6-mix buffer, which consisted of 1.37 mM citric acid, 0.46 mM ADA, 1.04 mM MOPS, 1.25 mM TAPS, 0.81 mM CHES, and 1.39 mM CAPS^51^.

### Absorption and CD spectra

The absorption spectra were measured with a UV-1800 spectrometer (Shimazu, Kyoto, Japan) at room temperature. The spectra contained scattering artifacts. Thus, the “net” absorption spectra were calculated by subtracting the scattering contributions, which were estimated by A + B/λ^4^ (λ in nm) as described^52^. The CD spectra were measured by a Jasco J-725 spectropolarimeter (Jasco, Tokyo, Japan) in the 300–700 nm region at 25 °C with a scanning speed of 200 nm/min. The accumulation was carried out eight times.

### Flash photolysis spectroscopy

Flash-induced absorbance changes were obtained in the 10 μsec to 10 sec time range on a single-wavelength kinetic system. For photoexcitation, the second harmonic (7 nsec, 532 nm) of a Q-switched Nd:YAG laser (Surelite I-10, Continuum, Santa Clara, CA) was used. The details have been previously described^53^. To improve the S/N ratio, 30–100 laser pulses were used at each measurement wavelength. The temperature was maintained at 25 °C. The GR concentration in all samples was 10 μM.

The H^+^ transfer reaction was measured with the pH indicator dye pyranine (Invitrogen, Tokyo, Japan). The procedure was described elsewhere^54^. Briefly, the flash-induced absorbance changes at 457 nm were measured before and after the addition of 100 μM pyranine. Then, the net signal from pyranine was calculated by subtraction. The nanodiscs were suspended in 0.5 mM MOPS, pH 7, containing 0.3 M NaCl.

### HPLC analysis and resonance Raman spectra

HPLC analysis was performed for both the dark- and light-adapted states. For dark adaptation, the nanodiscs were kept in the dark for 1 week in 6-mix buffer, pH 7, containing 0.3 M NaCl. For light adaptation, these samples were irradiated for 2 min by green LED light. The retinal extraction and the following HPLC analysis were performed as previously described^55^.

The resonance Raman spectra were measured by the homemade system described previously^45^. Briefly, the spectrometer is composed of a 0.5 m single spectrometer (Spex 500 M) equipped with a 1800 groove/mm holographic grating and a liquid-nitrogen-cooled UV-coated CCD detector (Spec-10:400B, Roper Scientific, Inc.). The 441.6 nm light (0.05 mW) from a helium–cadmium laser (IK5651R-G, Kimmon Electric Co., Ltd.) was used for the excitation light source. A Triax190 spectrometer (HORIBA Jobin Yvon) removed the excitation light, and the first order of the dispersed light by the 500 M spectrometer was imaged on the detector. All Raman spectra were measured at 25 °C with a single-beam method and calibrated using neat fenchone as a standard. Sample volumes were 100 μL and were contained in a quartz spinning cell (10 mm in diameter). The cell was spun at 1600 rpm. The nanodiscs were concentrated in 20 mM MOPS, pH 7, containing 0.3 M NaCl so that the GR concentrations became ~100 µM.

### Hydroxylamine bleaching and thermal denaturation

Both experiments were performed in the dark. For hydroxylamine bleaching, the reaction was started in pH 7 buffer (20 mM MOPS, 0.3 M NaCl) at 25 °C by the addition of concentrated hydroxylamine. Its final concentration was 50 mM. The thermal stability of GR was examined as described previously^56^. The nanodiscs were incubated in pH 8.5 buffer (50 mM HEPES, 0.3 M NaCl) at 65 °C. In both experiments, small aliquots were sampled at appropriate intervals and the absorbances at respective λ_max_ were measured after centrifugation (15000 rpm, 5 min at 4 °C) to remove the white aggregation. The initial absorbances at λ_max_ were 0.2 for hydroxylamine bleaching and 0.5 for thermal denaturation.

## Supplementary information

Supplementary Information
