# Supplementary material for: Functional importance of the oligomer formation of the cyanobacterial H+ pump Gloeobacter rhodopsin
Source: Sci Rep. 2019 Jul 24;9:10711. doi: 10.1038/s41598-019-47178-5 (PMC6656774; doi:10.1038/s41598-019-47178-5)
Supplement: Supplementary file 1 — Supplementary Information [file 41598_2019_47178_MOESM1_ESM.pdf]

Supplementary information

Functional importance of the oligomer formation of the cyanobacterial H^+ pump *Gloeobacter* rhodopsin

Azusa Iizuka[1], Kousuke Kajimoto[2], Tomotsumi Fujisawa[2], Takashi Tsukamoto[1,3], Tomoyasu Aizawa[1,3], Naoki Kamo[1], Kwang-Hwan Jung[4], Masashi Unno[2], Makoto Demura[1,3], Takashi Kikukawa[1,3,*]

[1]Faculty of Advanced Life Science, Hokkaido University, Sapporo 060-0810, Japan. [2]Department of Chemistry and Applied Chemistry, Faculty of Science and Engineering, Saga University, Saga 840-8502, Japan. [3]Global Station for Soft Matter, Global Institution for Collaborative Research and Education, Hokkaido University, Sapporo 001-0021, Japan. [4]Department of Life Science and Institute of Biological Interfaces, Sogang University, Seoul 04107, Republic of Korea.

*Corresponding Author
Tel.: +81-11-706-3435; Fax: +81-11-706-2771;
E-mail address: kikukawa@sci.hokudai.ac.jp
Postal address: Faculty of Advanced Life Science, Hokkaido University, Kita 10, Nishi 8, Kita-ku, Sapporo 060-0810, Japan

This file includes Supporting Figures S1 – S4.

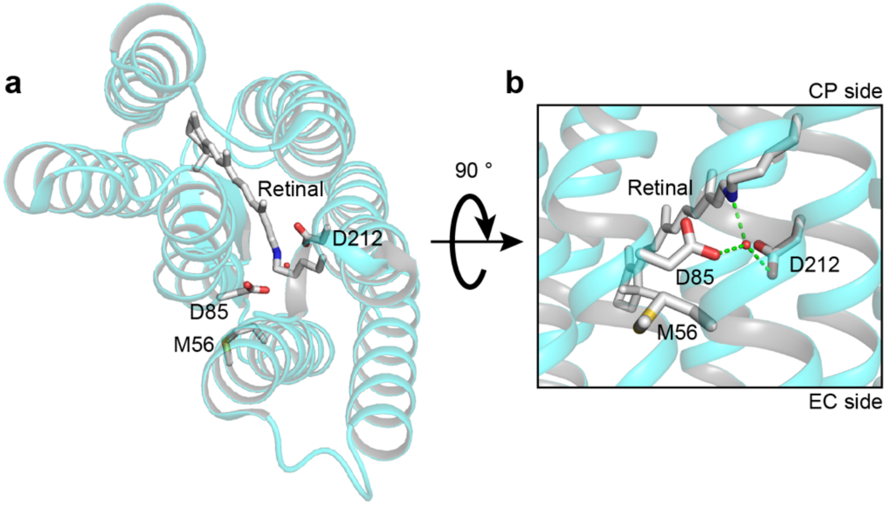

Figure S1. The positions of Met56, Asp85, and Asp212 residues in BR (PDB ID: 1C3W). They correspond to His87, Asp121, and Asp253 residues of GR. The top view from the CP side is shown in the left panel. Enlarged view of the H^+ acceptor region is shown in the right panel. The broken lines represent the proposed hydrogen bonds, and the red sphere represents water molecule.

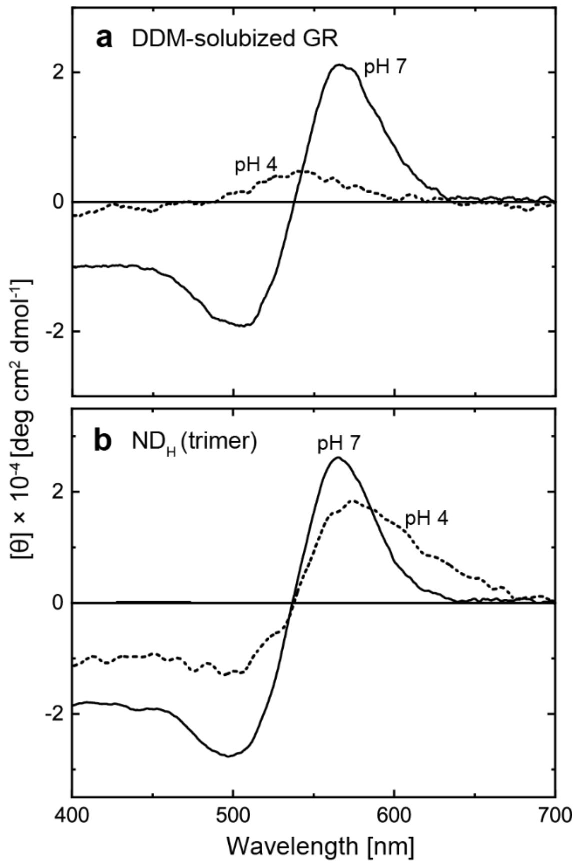

Figure S2. Comparison of CD spectra between GR in the DDM-solubilized state and the ND_H. The medium contained 6-mix buffer and 0.3 M NaCl. For DDM-solubilized GR, the medium was supplemented with 0.05% DDM. The pH was adjusted with HCl and NaOH.

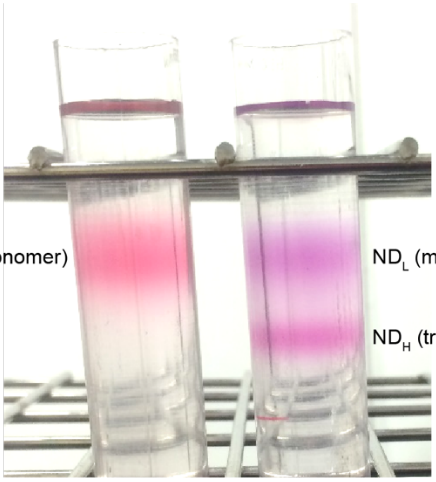

Figure S3. Bands of nanodiscs after sucrose density gradient centrifugation. The H87M shows a single band at almost the same position as ND$_L$.

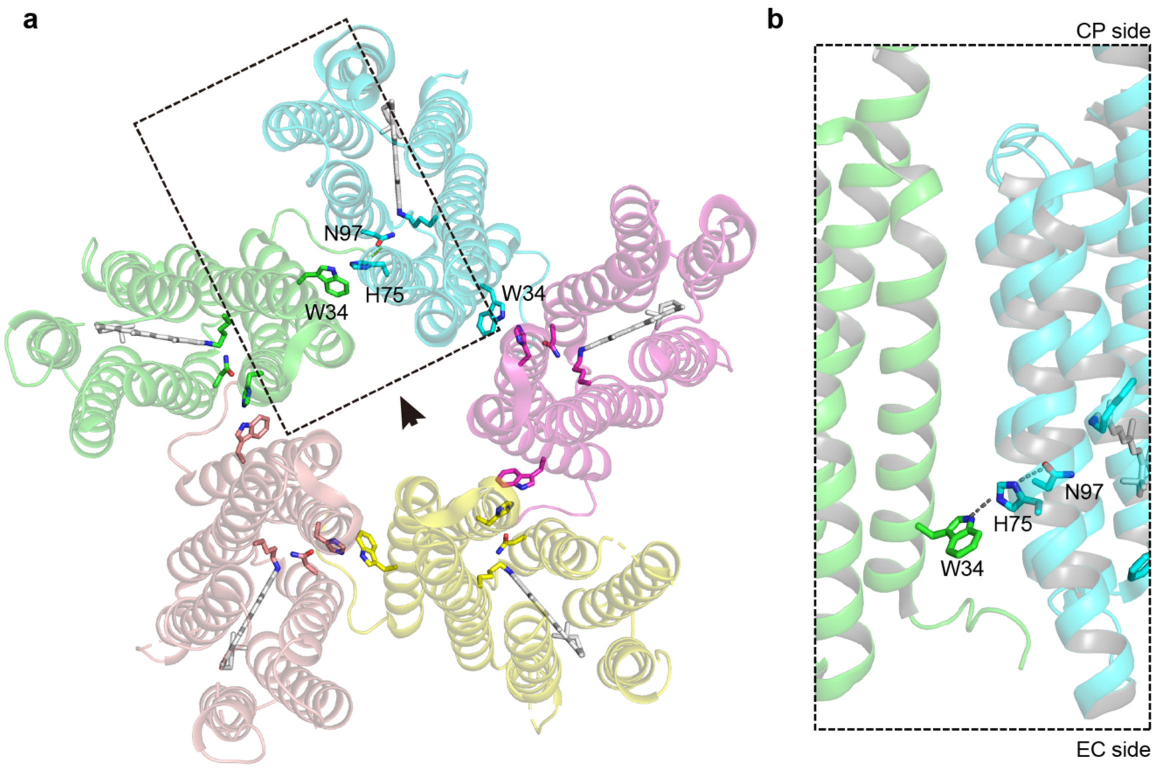

Figure S4. His-Asp and His-Trp interactions in BPR. These interactions in the BPR D97N mutant (PDB ID: 4KLY) are shown with the top view from the CP side (**a**) and the side view (**b**) of the broken square region in panel **a.**
